# Metastable Dihydrate of Sodium Chloride at Ambient Pressure

**DOI:** 10.1021/acs.jpclett.4c02752

**Published:** 2024-12-06

**Authors:** Rachael E. Hamp, Christoph G. Salzmann, Zachary Amato, Milz L. Beaumont, Hannah E. Chinnery, Peter Fawdon, Thomas F. Headen, Paul F. Henry, Liam Perera, Stephen P. Thompson, Mark G. Fox-Powell

**Affiliations:** † AstrobiologyOU, School of Environment, Earth and Ecosystem Sciences, Open University, Walton Hall, Milton Keynes MK7 6AA, United Kingdom; ‡ Department of Chemistry, University College London, 20 Gordon Street, London WC1H 0AJ, United Kingdom; § School of Physical Sciences, Open University, Walton Hall, Milton Keynes MK7 6AA, United Kingdom; ∥ ISIS Neutron and Muon Source, Rutherford Appleton Laboratory, Chilton, Didcot OX11 0QX, United Kingdom; ⊥ Department of Chemistry—Ångström Laboratory, Uppsala University, Box 523, 751 20 Uppsala, Sweden; # Diamond Light Source, Didcot OX11 0DE, United Kingdom

## Abstract

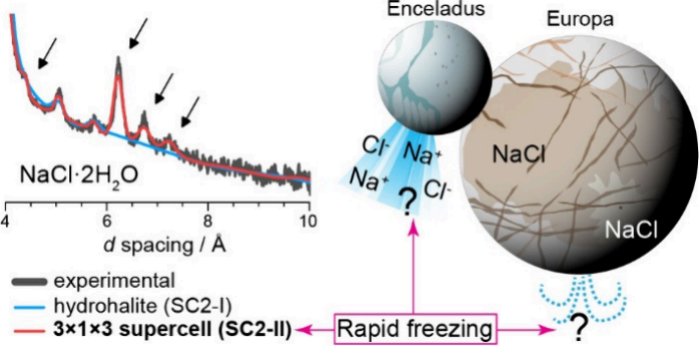

Sodium chloride (NaCl) plays an important role in geochemistry, biology, industry, and food production, and it is among the most common salts in the solar system. Here, we report the discovery of a metastable NaCl dihydrate formed through rapid freezing (10^1^–10^2^ K s^–1^) of a NaCl solution at ambient pressure. Using synchrotron X-ray and neutron powder diffraction, we show that it transforms irreversibly to hydrohalite and ice Ih above 190 K upon heating and propose it is structurally related to hydrohalite with a 3 × 1 × 3 supercell as its unit cell. Calorimetric analyses reveal that the new hydrate transforms to hydrohalite with a heat release of −3.47 ± 0.55 kJ mol^–1^. The identification of this new NaCl dihydrate on the surfaces of icy worlds such as the moons of Jupiter and Saturn could indicate regions of recent activity where subsurface brines have frozen rapidly, priority targets for upcoming planetary missions.

Sodium chloride (NaCl), commonly referred to as “table” or “rock” salt, is a naturally occurring ionic compound involved in a wide range of natural and industrial processes. NaCl plays a significant role in Earth’s geochemistry, it readily dissolves in water and is the most abundant salt in Earth’s oceans, regulating global ocean circulation and heat transport, and affecting the climate on a planetary scale.^1^ It is an essential component of global food supplies as both an ingredient and preservative, and it is widely used across many medical, agricultural, and industrial processes ranging from oil and gas extraction to chemical production and water softening. NaCl is also found across the Solar System, where it often provides evidence of liquid water processes.

Because of its importance in industrial and environmental processes, the thermodynamics of the NaCl-H_2_O system at elevated temperatures has been studied in depth, while the low temperature (<0 °C; 273.15 K) phase diagram has received comparatively less attention. Until recently hydrohalite (NaCl·2H_2_O; SC2-I), which can precipitate in sea ice on Earth,^2,3^ was the only known hydrate in the NaCl-H_2_O system, forming at temperatures below 273.25 K (at ambient pressure) (Figure 1). The ice Ih and SC2-I eutectic is reached at 23.3 wt % (5.20 mol kg^–1^) NaCl and 252 K at 1 bar. Recently, Journaux et al. identified two new NaCl hydrates (2NaCl·17H_2_O and NaCl·13H_2_O; SC8.5 and SC13, respectively) that form under high pressure (between 300 and 2,500 MPa; 3–25 kbar).^4^ Journaux et al. showed that once formed, SC8.5 can be recovered at ambient pressures and transitions to SC2-I and ice Ih upon warming at ∼235 K^4^. Extrapolation of the SC8.5 liquidus line indicates that SC8.5 should melt at a higher temperature than SC2-I and consequently, these authors postulated that SC8.5 should represent the thermodynamically stable form of NaCl-H_2_O below ∼235 K even at ambient pressure. The implication is that below this temperature SC2-I should convert to SC8.5.^4^ However, this transition has not yet been demonstrated in the laboratory, as the SC8.5 to SC2-I transition is not reversible upon further cooling. Cooling liquid solution at ambient pressure has thus far only produced SC2-I.

**Figure 1:**
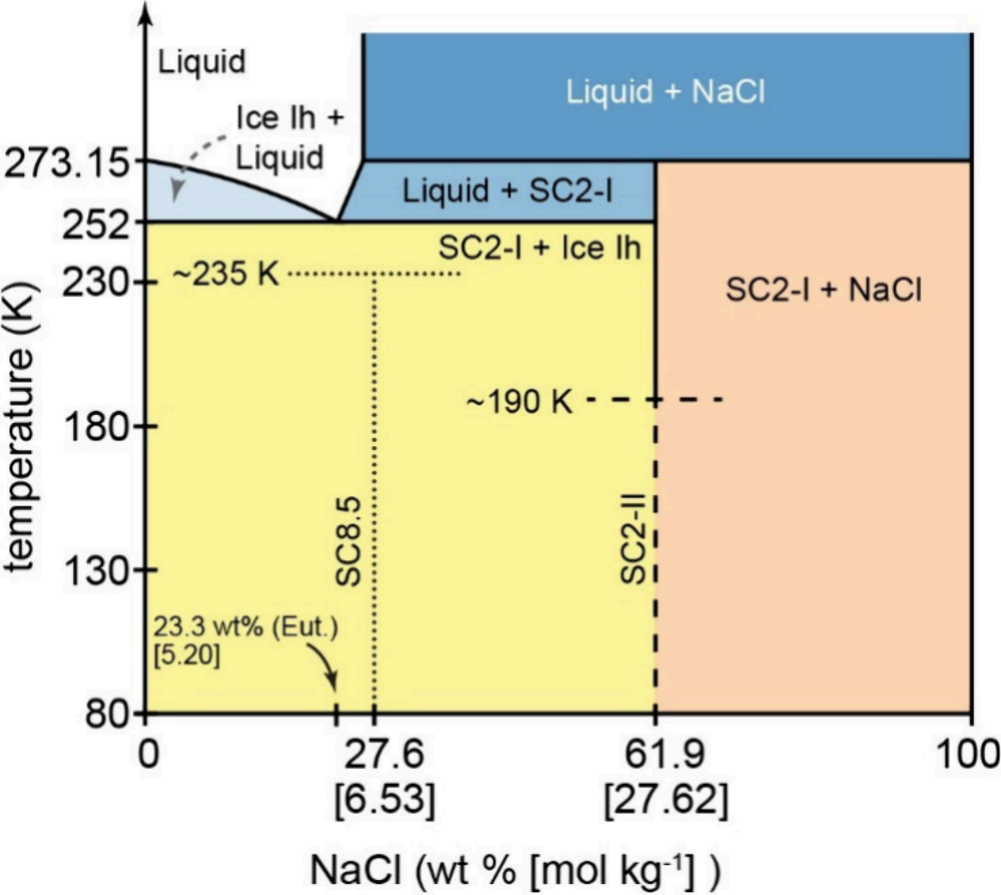
Phase diagram of the low-temperature NaCl-H_2_O system incorporating phase behavior of high-pressure hydrate 2NaCl·17H_2_O (SC8.5^4^) and proposed metastable dihydrate (SC2-II; this study). Dashed lines indicate stoichiometry and observed metastable transition temperatures of the new hydrate (SC2-II) to hydrohalite (SC2-I) and ice Ih at ∼190 K. Dotted lines indicate stoichiometry and phase transition temperature of SC8.5 to SC2-I and ice Ih at ∼235 K.^4^

In this work we report the formation of an additional NaCl dihydrate formed by rapid quench freezing of NaCl brines at ambient pressure to 77 K. To investigate the structure of the new NaCl hydrate, we used X-ray and neutron diffraction alongside Raman spectroscopy. Calorimetry was also used to study the phase change to SC2-I upon heating.

Samples were prepared by flash freezing (FF) solutions of NaCl in H_2_O or D_2_O to liquid nitrogen temperatures (see Experimental Methods) and were compared to solutions that were slow frozen (SF) (see Experimental Methods). A direct comparison of the FF and SF neutron diffraction data indicated the presence of a previously unidentified crystalline phase, forming only under the FF regime (Figure 2).

**Figure 2:**
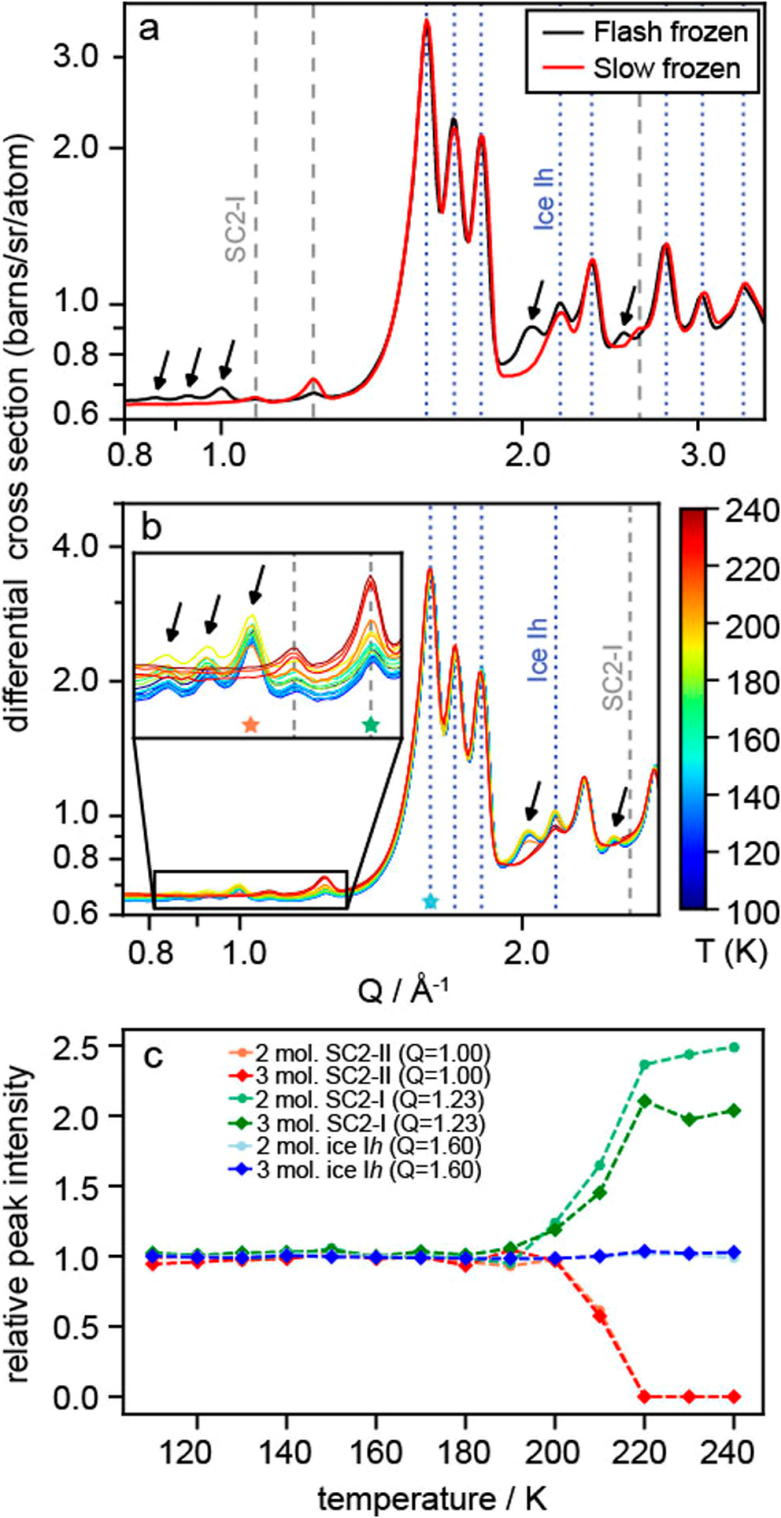
Neutron diffraction patterns from frozen 2 *m* NaCl solutions in D_2_O. Major SC2-I Bragg peaks are indicated with gray dashed lines, and ice Ih Bragg peaks, with blue dotted lines. New Bragg peaks are indicated by arrows. (a) Flash frozen sample (black) and a slow frozen sample (red) compared at 100 K where the presence of a new NaCl hydrate is exhibited in the FF sample. (b) Neutron diffraction patterns for a flash frozen sample being heated from 100 to 240 K, where the peaks for the new NaCl hydrate reduce in intensity upon heating and then disappear, while the peaks representing SC2-I increase within the same temperature interval. Red, green, and blue stars indicate the *Q* positions used to track peak intensities of SC2-II, SC2-I, and ice Ih, respectively, in panel c. (c) Changes in relative peak intensities of the Bragg peaks corresponding to SC2-II (red), SC2-I (green), and ice Ih (blue) for 2 and 3 m NaCl in D_2_O solutions. This shows the decrease and eventual loss of Bragg peaks for SC2-II, which is mirrored by the increase in SC2-I. However, the relative intensities of ice Ih peaks remain constant through heating.

Figure 2a displays neutron diffraction patterns at 100 K of a 2 m NaCl-D_2_O solution frozen at FF and SF conditions using the total scattering instrument Near and Intermediate Range Order Diffractometer (NIMROD) at the ISIS Neutron and Muon Source, U.K.^5,6^ The SF samples exhibit Bragg peaks for D_2_O ice Ih and SC2-I. In the FF sample, there are additional Bragg peaks, which do not correspond to any of the diffraction peaks produced by known phases in the NaCl-D_2_O system and therefore are indicative of a new crystalline material. Upon heating (Figure 2b), the additional Bragg peaks disappeared above 200 K and the intensity of the SC2-I peaks simultaneously increased, indicating an increase in the relative abundance of SC2-I in the sample concurrent with the disappearance of the new material. For SC2-I to increase in abundance, both NaCl and D_2_O must be liberated by the conversion, hence these data imply that the new crystalline material is a previously unknown hydrate of NaCl. To ascertain the hydration state of this new NaCl hydrate, we quantitatively analyzed the NIMROD neutron data. The constant intensity of the ice Ih peaks upon heating (Figure 2c) indicates that there was no net gain or loss of water molecules from ice during the decomposition of the unknown hydrate. The decomposition of a hydrate of hydration state >2D_2_O would release surplus D_2_O when converting to SC2-I, resulting in an increase in ice Ih in the sample and a corresponding increase in ice Ih Bragg peak intensity. Similarly, the decomposition of a phase of lower hydration state (<2D_2_O) would result in the consumption of D_2_O when forming SC2-I, leading to a reduction in ice Ih abundance and corresponding decrease in ice Ih Bragg peak intensity. Therefore, it was determined that the unknown NaCl hydrate must be a dihydrate, in order to conserve a constant proportion of ice Ih in the sample during warming. The new hydrate is named SC2-II to distinguish it from hydrohalite (SC2-I). The relative proportion of SC2-II decreased above 200 K, demonstrated by the increasing SC2-I: SC2-II peak-intensity ratio, and had disappeared entirely by 220 K.

To ascertain whether our FF samples contained only SC2-II and ice Ih, or a mixture of SC2-II, SC2-I, and ice Ih, cryo-Raman spectroscopy was conducted on a FF 2 m NaCl-H_2_O solution. Figure 3a shows an example of the Raman spectra from the O–H stretching region. Identical spectra were acquired from multiple locations across the sample, indicating a homogeneous distribution of phases within the sample. Figure 3a clearly indicates that both SF and FF spectra contain features diagnostic of ice Ih (e.g., at 3095, 3216, and 3325 cm^–1^). However, between 3350 and 3600 cm^–1^, clear differences can be observed between the FF and SF samples. The SF spectrum matches known SC2-I features^7^ with peaks at 3403, 3420, 3434, and 3537 cm^–1^, while the FF spectrum lacks these features and instead has prominent peaks at 3350, 3396, 3448, 3464, 3499 cm^–1^. These data reveal the location of spectral features that can be used to identify the new hydrate. Differences in peak position within the O–H stretching region imply differences in the vibrational environment of the water molecules between the two dihydrates, indicative of structural differences. The complete lack of SC2-I peaks demonstrates that our FF samples do not contain SC2-I and, and therefore must contain only SC2-II alongside ice Ih.

**Figure 3:**
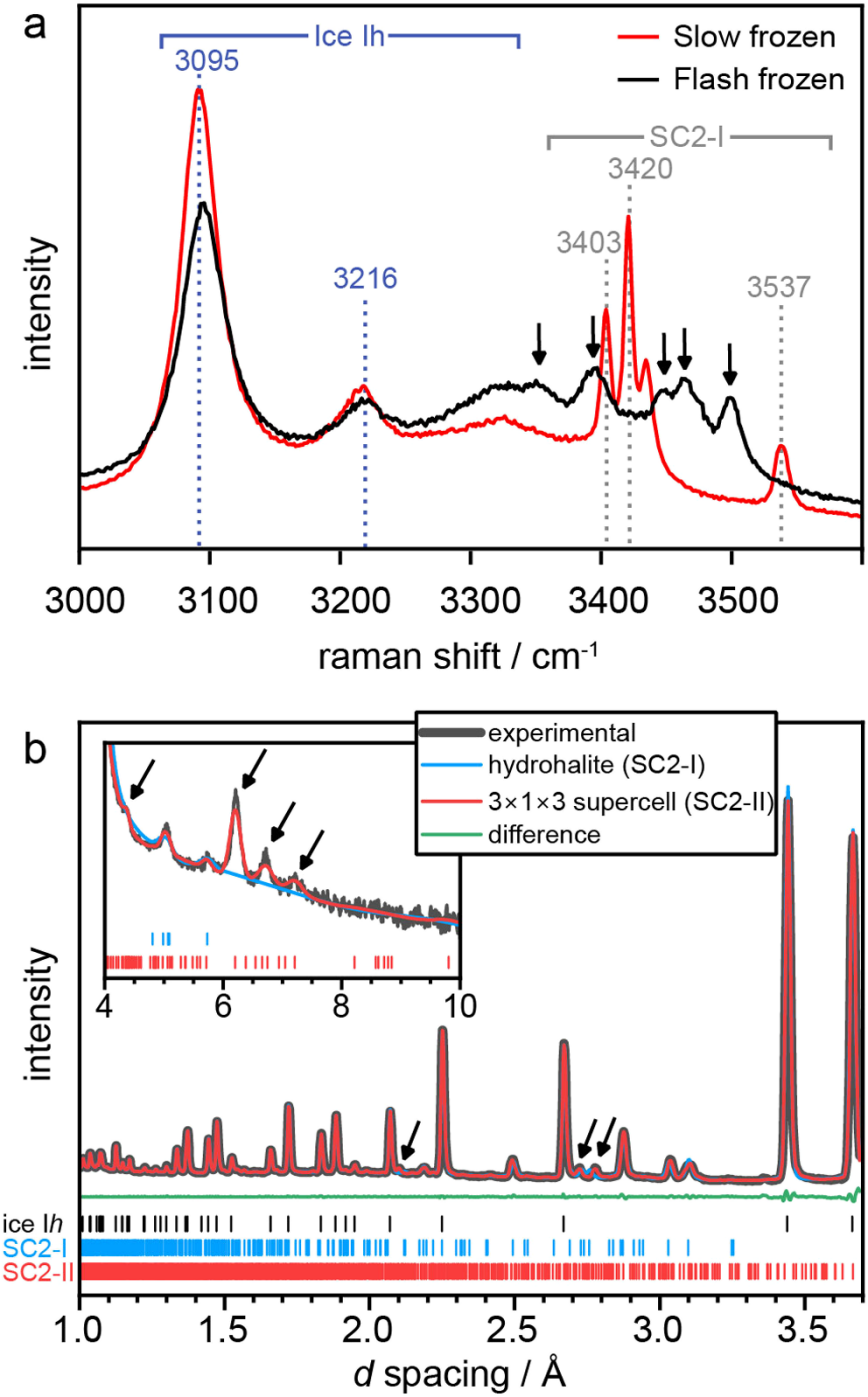
(a) Raman spectra of a 2 *m* NaCl-H_2_O solution both FF and SF. Spectra show the presence of water ice present in both samples; however, the FF sample has no peaks for SC2-I present, therefore concluding the FF sample does not contain any hydrohalite and it is solely SC2-II and ice Ih. Arrows indicate features characteristic of SC2-II at 3350, 3396, 3448, 3464, and 3499 cm^–1^. (b) POLARIS neutron diffraction patterns of a flash-frozen 2 *m* NaCl in D_2_O solution at 80 K. Structureless LeBail fits using the hydrohalite (SC-I) *P*2_1_/*c* structural model^10^ and a 3 × 1 × 3 supercell are shown in blue and red, respectively. The difference curve between the experimental data and the 3 × 1 × 3 supercell model is shown in green. The inset shows diffraction data at higher *d* spacing from a second detector bank. Regions where the SC-I model fails to describe the experimental features are indicated with arrows. Tick marks indicate the expected positions of Bragg peaks of (black) ice Ih, (blue) SC2-I, and (red) the 3 × 1 × 3 *P*2_1_/*c* supercell model.

To gain insights into the crystal structure of SC2-II, higher resolution diffraction patterns were obtained using the Polaris instrument at the ISIS Neutron and Muon Source.^8,9^Figure 3b shows the neutron diffraction patterns of a FF sample at 80 K. Many Bragg peaks in locations typical of SC2-I are observable, alongside unknown Bragg peaks. However, because our Raman data show that flash freezing produces a sample devoid of SC2-I, containing only ice Ih and a new hydrate (Figure 3a), a single structural model representing a single crystalline phase must explain all the observed nonice Ih Bragg peaks, including the unknown peaks and those typical of SC2-I. Therefore, the diffraction data was first fitted with the LeBail method using the structural model of SC2-I with *P*2_1_/*c* space-group symmetry.^10^ The fit can describe many of the observed diffraction features indicating a structural relationship between SC2-I and SC2-II. Yet, the arrows in Figure 3 indicate diffraction features that could not be reproduced with the SC2-I structural model. Degrading the *P*2_1_/*c* space-group–space-group symmetry to *P*1̅ lead to improvements fitting the features at around 2.1 and 2.7 Å. Yet, the high *d* spacing features above 6 Å, previously identified in the NIMROD data at low *Q* values (Figure 2), could not be described, indicating that a larger unit cell is required. While staying with *P*2_1_/*c*, supercells were explored systematically with LeBail fits, which showed that a 3 × 1 × 3 supercell reproduces the three diffraction features above 6 Å. Tripling the cell edges are klassengleiche subgroups of *P*2_1_/*c*. The corresponding unit cell parameters are *a* = 19.0882(10) Å, *b* = 10.1265(4) Å, *c* = 19.6776(13) Å, and β = 116.038(4)°. Given that SC2-II was the minority component besides ice Ih in our samples, it was not possible to solve the structure of SC2-II, hence the 3 × 1 × 3 supercell is the current best explanation for the data. Other possible structures, in particular larger supercell structures of multiples of the unit cell parameters, cannot be ruled out. Yet, given the similar diffraction properties, and the absence of SC2-I as revealed by Raman, it is fair to assume that the SC2-II structure may be a related, perhaps distorted or rearranged, version of SC2-I.

To ensure the behavior observed in the NaCl-D_2_O system is applicable to the NaCl-H_2_O system, both H_2_O and D_2_O samples were analyzed using synchrotron X-ray powder diffraction (pXRD) on beamline I11 at Diamond Light Source, Oxford, U.K.^11,12^ The frozen H_2_O and D_2_O solutions produce nearly identical pXRD patterns (Supporting Information Figure S1), therefore giving us confidence that the results observed in the neutron data for a NaCl-D_2_O system are applicable to the NaCl-H_2_O system and ensuring the isotopic differences do not affect the formation of SC2-II.

It is fundamental to assess the stability of SC2-II with varying physical conditions, such as temperature. The NIMROD neutron data provided a guide as to the temperature of the transition from SC2-II into SC2-I, however, the data was collected at 10 K intervals, and therefore the precise start and end temperatures of the transitions between the different NaCl dihydrates are not well-defined. To determine the transition temperatures more accurately, we conducted heating experiments using capillary-loaded samples in a nitrogen cryostream and monitored by pXRD (details in Experimental Methods).

Freezing a 2 *m* NaCl-H_2_O solution in a 1 mm polyimide (Kapton) capillary produced the anticipated pXRD pattern, with Bragg peaks for ice Ih and SC2-II present at 100 K. All samples show one phase transition upon heating as SC2-II decomposes to give SC2-I. The heating profile from 170 to 206 K for the 2 *m* NaCl-H_2_O sample is shown in Figure 4. We note the same heating profile and phase transition is also generated for the 2 *m* NaCl-D_2_O sample (Figure S2). As the temperatures exceeded 192 K, intensities for unique SC2-II Bragg peaks decreased, while simultaneously the peaks shared by SC2-I and SC2-II increased in intensity. Upon heating to 204 K none of the diagnostic SC2-II peaks remained, indicating the sample had fully transitioned from SC2-II to SC2-I. An extended range pXRD pattern for the FF NaCl-H_2_O samples is presented in Figure S2, which shows the high *d* spacing diffraction features observed on the NIMROD and Polaris instruments. We note that in pXRD experiments, the coproduction of both SC2-I and SC2-II cannot be ruled out in the FF samples. This is because the flash freezing of samples was conducted in a capillary tube (see Experimental Methods), leading to the potential for different freezing conditions compared with samples analyzed by neutrons or Raman. For this reason, we use pXRD to indicate the presence or absence of SC2-II only.

**Figure 4:**
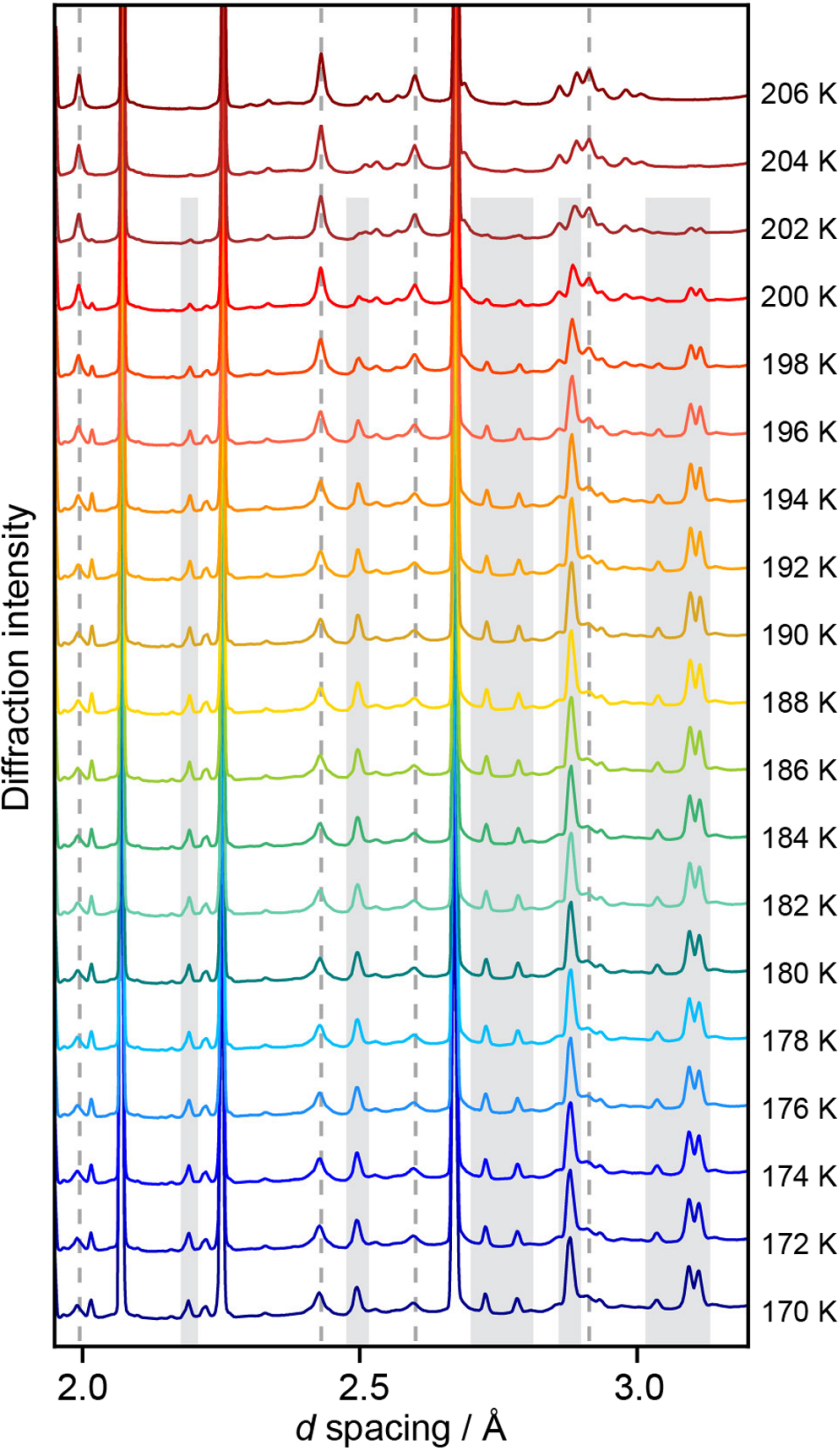
pXRD heating profile from 170 to 206 K for an FF 2 *m* NaCl solution, where SC2-II transitions into SC2-I during the heating cycle. Shaded gray regions show major Bragg peak locations indicative of SC2-II. Dashed lines indicate Bragg peak locations shared by SC2-II and SC2-I.

Having determined the temperature range for the transition of SC2-II into SC2-I, differential scanning calorimetry (DSC) was carried out to determine the enthalpy change of this transition (see Experimental Methods). The DSC scans showed an exothermic transition at an onset temperature of 190 K with heat release of −3.47 ± 0.55 kJ mol^–1^, which is in line with the transition from SC2-II into SC2-I observed in pXRD (Figure 4). Furthermore, the phase transition was not observed upon second heating after recooling the sample to 100 K at a rate of 50 K min^–1^, indicating that the transition from SC2-II to SC2-I is irreversible. The exothermic and irreversible phase transition indicates that SC2-II is metastable with respect to SC2-I. As for all metastable materials, the onset temperature of the transition to the stable phase will be time dependent meaning that prolonged annealing at temperatures below the onset temperature from the DSC measurements should still lead to transformation.

This study has expanded on the knowledge of the low-temperature phase diagram of NaCl-H_2_O (Figure 1). The identification of SC2-II, that forms at ambient pressure through rapid freezing, marks the first new hydrate of NaCl that forms from liquid solution at ambient pressure to be discovered for 200 years. SC2-II is metastable, transforming to SC2-I through an exothermic reaction, at temperatures above 190 K (Figure 5). Other metastable transitions are known to occur in the NaCl-H_2_O system; for example, upon warming, a solid mixture of ice Ih and anhydrous NaCl converts irreversibly to SC2-I and ice Ih at ∼243 K;^13^ however, SC2-II is the first identified metastable hydrate of NaCl.

**Figure 5:**
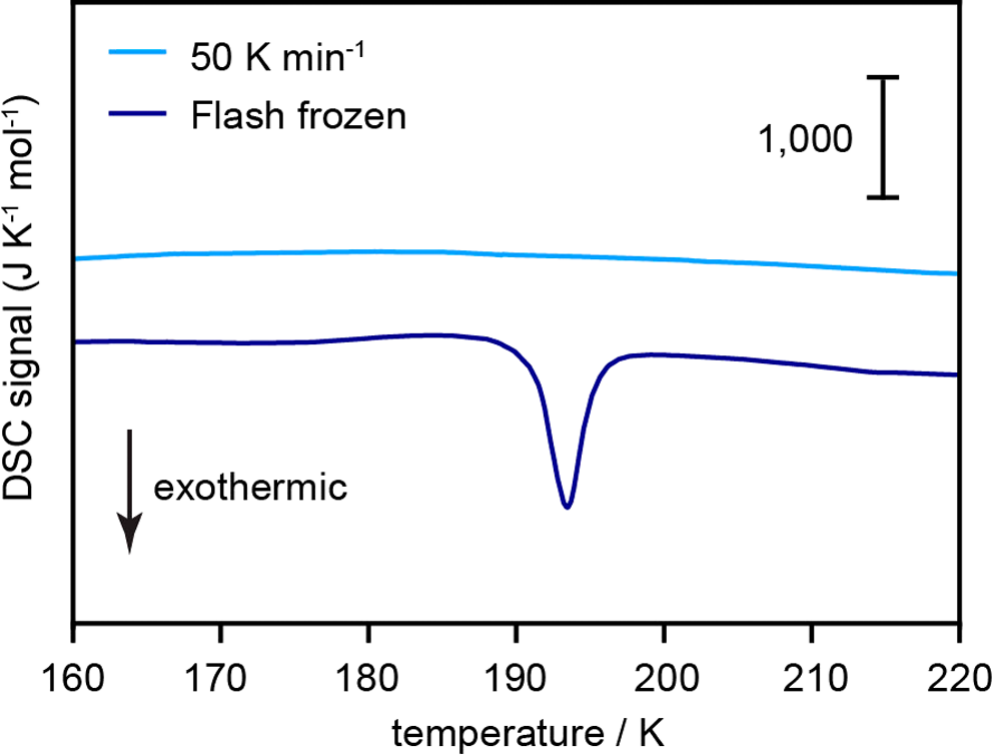
DSC heating scans for two FF 2 *m* NaCl-H_2_O fluids frozen showing the exothermic transition from SC2-II to SC2-I at an onset temperature of approximately 190 K. DSC heating scans for the same 2 *m* NaCl-H_2_O samples refrozen at a rate of 50 K min^–1^ are also shown to highlight that SC2-II does not reform upon freezing at a slower rate.

Because SC2-II did not form at slower cooling rates, it is clear that there is a strong kinetic control on its formation, whereby at slower cooling rates SC2-I is favored. The formation of SC2-II from liquid solution must depend on the degree to which SC2-I precipitation is inhibited, since the growth of SC2-I crystals will deplete the solution in Na^+^ and Cl^–^ ions, preventing further hydrate precipitation. In our samples, hydrate precipitation is likely occurring within a network of narrow brine-filled channels at boundaries and triple-junctions between crystals of ice Ih, which is rapidly established even at these fast-cooling rates.^14,15^ These brine channels will be characterized by rapidly increasing salinity alongside decreasing temperature. The kinetics of SC2-I precipitation under such conditions are an understudied area. Homogenous nucleation rates of SC2-I at a constant temperature of −18 °C (255.15 K) have been observed to be particularly sluggish, on the order of days.^16^ These authors speculate that the observed slow nucleation is the result of an unknown metastable prenucleation phase which inhibits SC2-I formation by increasing the nucleation barrier and/or decreasing supersaturation. As a metastable dihydrate of NaCl, SC2-II is a candidate for such a parent phase to SC2-I, which may, under slower cooling conditions, relax readily into SC2-I. We note that at the quench cooling rates examined here (10^1^ – 10^2^ K s^–1^), SC2-II exclusively forms, indicating that regardless of initial nucleation, growth of SC2-I has been entirely inhibited. Determining the cooling rate at which SC2-I formation is bypassed is an area of ongoing focus.

While it is unlikely that SC2-II could form on Earth through natural processes as the required freezing rate is too fast for any natural system, there may be implications for industrial processes, particularly cryogenic preservation of food and other materials. Quench-freezing materials in liquid nitrogen is a common technique across many industries, and, based on our findings, if NaCl is present, there is a strong possibility of forming SC2-II. The latent heat release upon SC2-II conversion to SC2-I above 190 K could have implications for cryo-preserved sample integrity.

Beyond Earth, there are locations in the outer Solar System where SC2-II may be prevalent, such as icy worlds that contain salty subsurface oceans. Ocean-bearing icy worlds, which include a growing number of the icy satellites of the giant planets as well as dwarf planets such as Ceres, are predominantly airless bodies that contain water oceans overlain with an ice crust, often with very cold surface temperatures (≤100 K). Some host ’cryovolcanic’ processes, where liquid water is rapidly emplaced to their surfaces, leading to freezing rates plausibly comparable to this study. Importantly, these icy bodies are also known to contain liquid water bearing Na^+^ and Cl^–^ in their subsurface: NaCl has been identified in the plumes of Enceladus, a moon of Saturn,^17^ at Europa, a moon of Jupiter^18,19^ and on the surface of Ceres, a dwarf planet present in the main asteroid belt.^20^ In all cases, detections of NaCl are best explained by the freezing of subsurface salty liquid water. However, the host phases of NaCl at these worlds is not clear. At Ceres, SC2-I has been identified through near-infrared reflectance.^20^ But data from the much colder Europa, a moon of Jupiter, does not show evidence of SC2-I.^21−25^ NaCl at Europa is instead inferred through observations of NaCl radiation products.^18,19^ The identification of SC2-II adds to the recently discovered high-pressure hyperhydrates^4^ in increasing the possible diversity of NaCl hydrates that could exist on Europa’s surface. Because SC2-II forms from liquid solutions at low pressures, it could feasibly form directly through cooling of ocean water at the surface or within the shallow ice shell of Europa and other icy worlds, such as within plumes or resurfaced regions. The presence of SC2-II on icy worlds would indicate rapid freezing of brines, acting as a marker for materials from plumes, jets or other rapid resurfacing events that will be of great interest to upcoming missions such as NASA’s Europa Clipper and ESA’s JUpiter ICy Moons Explorer (JUICE).

Based on the transition temperatures of SC2-II into SC2-I (∼190 K), it can be predicted that SC2-II could be present on the surface of an icy world if the immediate environment does not experience heating events over this temperature. The global mean annual temperature on Europa is approximately 90 K^26^ and the surface of Enceladus at low latitudes during the day is approximately 80 K,^27^ therefore, SC2-II could exist on the surface of these icy worlds unless they were to experience a heating event, such as an impact. However, as a metastable phase, it is thermodynamically favorable for SC2-II to convert over time to SC2-I. The presence of SC2-II on the surface of icy worlds could, in principle, be used to indicate a recent emplacement of brines to the surface, because older deposits may have transitioned to SC2-I. This is important for future missions, as this material will be geologically recent and will have experienced less alteration and will therefore likely be the most pristine representation of a subsurface liquid water reservoir on the surface of these icy worlds. Future work should aim to characterize the temperature-dependent kinetics of the SC2-II to SC2-I conversion at icy world surface conditions.

Finally, the energy released through the decomposition of SC2-II into SC2-I has important ramifications for icy worlds. In certain settings, such as locations of concentrated brine, If SC2-II were to transition into SC2-I the energy released (−3.47 ± 0.55 kJ mol^–1^) could melt surrounding water ice. The energy required to melt one mole of ice Ih is 6.02 kJ mol^–1^ and therefore when the mole fraction of SC2-II exceeds 1.8 with respect to ice Ih, this would cause local melting of ice if it were to undergo a transition into SC2-I. This may have significance for geological processes in the upper ice shells of icy worlds.

This study has identified a new NaCl hydrate, SC2-II, a metastable dihydrate with a proposed 3 × 1 × 3 *P*2_1_/*c* supercell structure, formed through rapid freezing of NaCl solutions at ambient pressure. SC2-II is metastable with respect to SC2-I, releasing latent heat as it decomposes to SC2-I and ice Ih above ∼190 K. These features set SC2-II apart from other known hydrates of NaCl. This is only the third new hydrate of NaCl to be discovered in over 200 years and the only other NaCl hydrate besides SC2-I known to form directly from liquid at ambient pressures. Our findings contribute to the growing recognition of the structural diversity and phase behavior complexity in the low-temperature NaCl-H_2_O system.

## Experimental Methods

### Sample Preparation

#### NaCl Solution Preparation

NaCl solutions were made with varying concentrations. NaCl (BioXtra Brand, reagent grade, >99.5% pure) was dissolved in ultrapure water (Milli-Q) or deuterated water, at concentrations of 0, 2, 3, and 5.2 *m*.

#### Experimental Freezing

Frozen samples were made via two freezing regimes. Slow frozen samples were produced by placing 5 mL of room-temperature NaCl solution into a freezer in a sealed container at −20 °C (253.15 K) for 1 h, before being transferred to a −80 °C (193.15 K) freezer for a minimum of 12 h. Beginning at 20 °C and becoming equilibrated to −20 °C after 300 min resulted in a maximum average cooling rate of ∼0.67 K min^–1^ (∼0.01 K s^–1^), which is consistent with the cooling rate measured during a similar preparation method in Fox-Powell and Cousins.^15^ Unless otherwise stated, flash frozen samples were made through two methods: (i) by dripping sample directly into liquid nitrogen (droplet size range of 1–10 mm), which produced an approximate freezing rate of 10^1^–10^2^ K s^–1^^15^ (used for NIMROD and DSC); or (ii) by dripping liquid sample (droplet size of 10 μL) directly onto an aluminum plate held at liquid nitrogen temperature (used for Polaris samples). Based on prior work,^28^ the freezing method of (ii) will produce a faster rate for a given droplet size than (i); thus, we consider 10^1^–10^2^ K s^–1^ as a minimum.

### Neutron Diffraction

#### Near and Intermediate Range Order Diffraction (NIMROD)

Experiments were conducted at ISIS Neutron and Muon Source, Harwell, Oxford, U.K. NIMROD analysis under optimal conditions can obtain structural information on continuously probed length scales ranging from 0.01–100 Å^–1^. For all analysis conducted on NIMROD the beam size was 22 mm × 26 mm; this ensured a large cross-section of the sample was analyzed, generating diffraction patterns that were representative of the bulk ice grain population.

#### NIMROD Sample Preparation

NaCl bearing deuterated ice samples (as prepared above) were crushed through a sieve under liquid nitrogen to obtain a grain size of <250 μm. Crushed samples were loaded, under liquid nitrogen, into a flat plate vanadium sample foil holder containing approximately 2 mL of sample. Sample was loaded into the neutron beam (path length ranging from 1 to 2 mm) under He environment with a pressure of 30 mbar and a temperature of 100 K, with cooling supplied via a 4 K cryostat. Crushed samples were used for the analysis to ensure a homogeneous sample and minimize any effect of preferential crystal orientation.

#### NIMROD Experiment and Heating Experiments

A scan was carried out at a temperature of 100 K to collect an accumulated proton-beam current to target of 40 μA. Samples were then heated in situ from 100 to 240 K at a rate of 1 K min^–1^, with a scan (10 μA) carried out at each 10 K step. Initial calibration and data reduction were conducted using GudrunN software.^29^ The raw data from the NIMROD neutron scattering experiments were merged for all detectors and normalized on a per atom basis.

#### Polaris (High Intensity, Medium Resolution Powder Diffractometer) Diffraction

Experiments were conducted at ISIS Neutron and Muon Source, Harwell, Oxford, UK. For all analysis conducted on Polaris, sample was contained in 8 mm cylindrical vanadium sample holders and analyzed with a beam size was 40 mm × 15 mm, this ensured a large cross-section of the sample was analyzed, generating diffraction patterns that were representative of the bulk ice grain population.

#### Polaris Sample Preparation

NaCl bearing deuterated ice grains (as prepared above) were crushed under liquid nitrogen in a pestle and mortar. Crushed samples were loaded, under liquid nitrogen, into a cylindrical vanadium sample holder containing approximately 2 cm^3^ of sample. Crushed samples were used for the analysis to ensure a homogeneous sample and minimize any effect of preferential crystal orientation. Sample was loaded into the neutron beam under He environment with a pressure of 100 mbar and a temperature of 80 K.

#### Polaris Experiment

Neutron diffraction was monitored at a temperature of 100 K. A continuous scan was run until an accumulated current 850 μA had been collected. The detailed neutron diffraction analysis was carried out using the GSAS software^30^ by refining structural models to fit the diffraction data.

### X-ray Diffraction

#### High-Resolution Powder X-ray Diffraction (pXRD)

Experiments were conducted on the I11 beamline at the Diamond light source in Harwell, Oxford, U.K.

#### Sample Preparation

NaCl-H_2_O and NaCl-D_2_O of varying concentrations were loaded into Kapton tubes (1 mm in diameter). These were plunged into liquid nitrogen to rapidly freeze. Samples were then loaded onto the pXRD, and a nitrogen cryostream set to 80 K was placed over the sample.

#### pXRD Experiment and Heating Experiments

The instrument was calibrated using NIST Si reference powder SRM640c. A 1-h high-resolution 45 multianalyzing crystal (MAC) detector system (MAC wavelength = 0.824167; zero point = +0.076803) scan was conducted on each sample at 100 K to observe the phase assemblage in each of the flash frozen NaCl-H_2_O and NaCl-D_2_O samples. The temperature for all heating experiments was controlled by a nitrogen cryostream flowing over the sample, providing fine control on the temperature heating cycles. The temperature of the cryostream was raised to 170 K, before the known transition temperature for SC2-II into SC2-I. The sample was then heated from 170 to 210 K at a rate of 1 K min^–1^, with a position sensitive detector (PSD wavelength = 0.824295; zero point = −0.00769) scan taken every 2 K to determine the transition temperatures between phases.

### Cryo-Raman Spectroscopy

Raman analysis was conducted using a FDCS196 Linkam cryostage connected to a Horiba LabRAM HR Evolution Raman Spectrometer. Flash frozen NaCl-H_2_O samples were prepared by dropping 10 μL of a 2 *m* NaCl-H_2_O solution onto a glass slip on a precooled stage, set to 77 K. Slow frozen NaCl-H_2_O samples were prepared by dropping 10 μL of a 2 *m* NaCl-H_2_O solution onto a glass slip at room temperature and was cooled to 77 K at a rate of 20 K/min. Analysis of both the FF and SF samples were conducted at 100 K using the 532 nm laser.

### Differential Scanning Calorimetry (DSC)

Flash frozen NaCl-H_2_O samples (using method Ice Grain Production Method) were loaded into stainless-steel capsules under liquid nitrogen and transferred into a precooled advanced double-furnace DSC 8000 from PerkinElmer. The furnace temperature was held for 4 min at 95 K allowing for equilibration and then heated to 293 K at a rate of 10 K min^–1^. To investigate the effect of a slower freezing rate, following initial heating, a molten sample was cooled back to 95 K at a rate of 50 K min^–1^ and then held at 95 K for 4 min to allow for the temperature to equilibrate. The sample was heated to 293 K at a rate of 10 K min^–1^.

## Supporting Information Available

pXRD comparisons of flash-frozen (FF) 2 m NaCl-H_2_O and 2 m NaCl-D_2_O solutions; pXRD pattern of FF 2 m NaCl-H_2_O solution (region for high *d*-spacing features indicative of SC2-II); pXRD heating profile for FF 2 m NaCl-D_2_O solution SC2-II to SC2-I transitions) (PDF)
