# Supplementary material for: Metastable Dihydrate of Sodium Chloride at Ambient Pressure
Source: J Phys Chem Lett. 2024 Dec 6;15(50):12301–8. doi: 10.1021/acs.jpclett.4c02752 (PMC11664646; doi:10.1021/acs.jpclett.4c02752)
Supplement: Supplementary file 1 — jz4c02752_si_001.pdf [file jz4c02752_si_001.pdf]

Metastable dihydrate of sodium chloride at ambient pressure

Authors: Rachael E. Hamp[a]*, Christoph G. Salzmann[b], Zachary Amato[c,d], Milz L. Beaumont[b], Hannah E. Chinnery[c], Peter Fawdon[c], Thomas F. Headen[d], Paul F. Henry[d,e], Liam Perera[f], Stephen P. Thompson[f] and Mark G. Fox-Powell[a]

Author Address: [a] AstrobiologyOU, School of Environment, Earth and Ecosystem Sciences, Open University, Walton Hall, Milton Keynes MK7 6AA, UK.

[b] Department of Chemistry, University College London, 20 Gordon Street, London WC1H 0AJ, UK

[c] School of Physical Sciences, Open University, Walton Hall, Milton Keynes MK7 6AA, UK

[d] ISIS Neutron and Muon Source, Rutherford Appleton Laboratory, Chilton, Didcot, OX11 0QX, UK

[e]Department of Chemistry - Ångström Laboratory, Uppsala University, Box 523, 751 20, Sweden

[f]Diamond Light Source, Didcot, OX11 0DE, UK

*Corresponding author: Rachael Hamp (rachael.hamp@open.ac.uk)

Supporting Information:

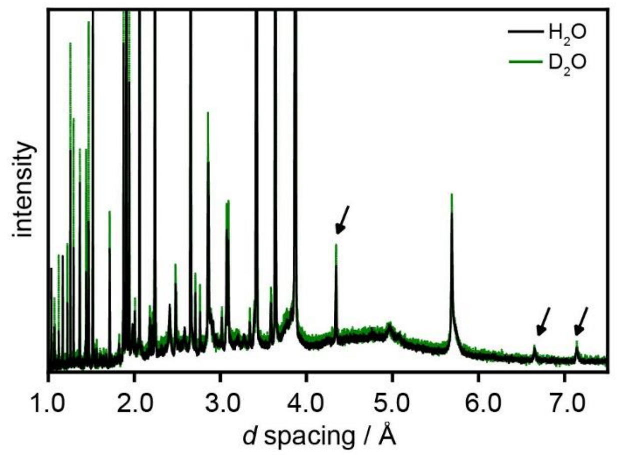

Figure S1: *pXRD pattern for a flash frozen 2 m NaCl-H_2O solution (black) and a flash frozen 2 m NaCl-D_2O solution containing crystalline SC2-II and Ice I*h. *Arrows indicate high* d*-spacing Bragg peaks diagnostic of SC2-II.*

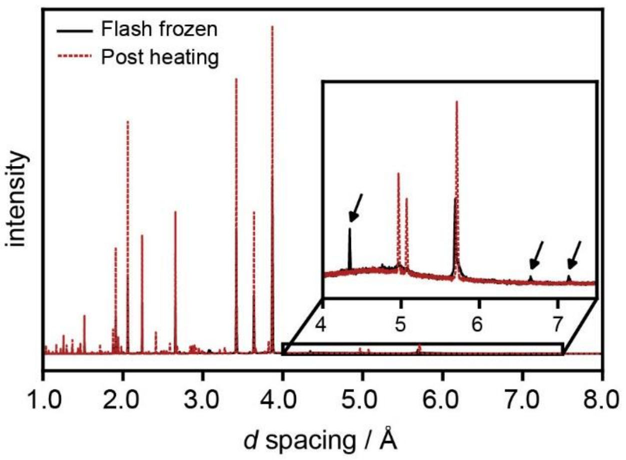

Figure S2: *pXRD pattern for a flash frozen 2 m NaCl-H_2O solution with insert showing the high* d*-spacing peaks that are indicative of SC2-II.*

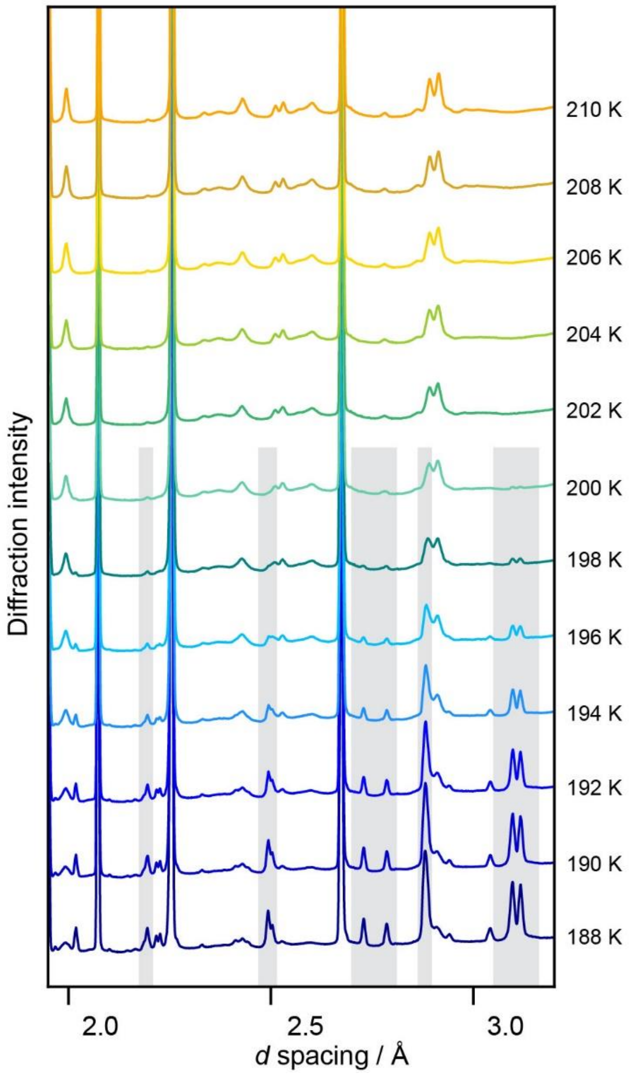

Figure S3: *pXRD heating profile from 188 K to 210 K for a flash frozen 2 m NaCl-D_2O solution that contains SC2-II, and Ice I*h*, where SC2-II transitions into SC2-I during the heating cycle. Shaded gray regions indicate major Bragg peak locations indicative of SC2-II.*
